# Relationship between self-rated health, sense of coherence and physical activity in a survey of secondary school students: A correlational study

**DOI:** 10.1038/s41598-025-12686-0

**Published:** 2025-07-22

**Authors:** Ilona Karácsony, Gabriella Hideg-Fehér

**Affiliations:** 1 Faculty of Health Sciences, Institute of Basic Health Sciences, Midwifery and Health Visiting, Department of Obstetrics and Prevention, University of Pécs, Campus Szombathely, Pécs, Hungary; 2 Faculty of Health Sciences, Institute of Physiotherapy and Sport Sciences, Department of Sport Science, University of Pécs, Pécs, Hungary

**Keywords:** Physical activity, The sense of coherence, Secondary school students, Vitality and mental health, Preventive behaviours, Health care, Quality of life

## Abstract

The aim of the present research was to assess the physical activity of secondary school students and to understand its effect on subjective general health, vitality and mental health. A quantitative cross-sectional study was conducted among adolescents aged 15–19 (*n* = 448) years attending a full-time secondary school. The paper-based questionnaire included self-reported questions on physical activity in addition to socio-demographic variables, while the standardised instrument measured dimensions of subjective health (Health Thermometer - EuroQol scale, EQ VAS, Health Survey Short Form SF-36 Questionnaire) and Sense of Coherence (SOC 13). The amount of physical activity was adequate for 22.2%. Regular physical activity had a positive effect on self-reported health perception (*p* < 0.05). The mean scores for mental health, vitality and general health perception were significantly higher among students who were adequately or nearly adequately physically active (*p* < 0.05). A positive correlation was found between sense of coherence and frequency of physical activity (*p* < 0.05). The means of the self-reported health indicators and the sense of coherence were almost identical between the groups (*p* > 0.05). The sense of coherence plays a role in the changes in the regularity of physical activity, and a linear stochastic relationship between the variables was demonstrated.

## Introduction

According to WHO estimates, there are between 4 and 5 million preventable deaths worldwide each year^1^. Regular physical activity is an important part of healthy lifestyle and can play a key role in maintaining and improving the health of the population. Physical activity is key to the primary prevention and management of chronic non-communicable diseases (NCDs) (including mental health conditions, cardiovascular disease, type 2 diabetes mellitus, obesity, musculoskeletal conditions and cancer). Physical activity also has a positive effect on mental health, reducing symptoms of depression and anxiety, improving thinking skills, preventing deterioration in brain function and reducing the risk of developing dementia and Alzheimer’s disease^2^. Regular physical activity of adequate quality and quantity has both preventive and curative benefits, with physical, psychological, social, environmental and protective effects^2,3^. The global epidemic of physical inactivity is associated with a range of chronic diseases and premature mortality, which represents a significant economic burden in addition to the disease burden. Physical inactivity is also associated with direct health costs, including time lost from work^4^.

The physiological and psychological benefits of physical activity are well documented, but the majority of adolescents are still not engaging in sufficient time and intensity of physical activity. According to WHO recommendations, children and adolescents should engage in at least 60 min of moderate-intensity (preferably aerobic) physical activity per day and three times per week of vigorous-intensity and muscle-strengthening physical activity^5^. Estimates suggest that more than 80% of adolescents fail to meet these standards^6^. According to the pooled results of the 2018 HBSC (Health Behaviour in School-aged Children) survey, less than one in five adolescents meets WHO recommendations for physical activity, with only 19% of adolescents doing 60 min of moderate-intensity physical activity per day. Since 2014, the proportion of regular physical activity has decreased in about a third of the countries surveyed, particularly among boys, but the proportion of boys is still higher, with almost half of boys (49%) and a third of girls (35%) doing vigorous physical activity four or more times a week. The gender gap with increasing age is also present for moderate (aerobic) and vigorous intensity exercise^7^. Following a systematic review of the literature (*n* = 43) from 1969 to 2018, Pinto et al.^8^ found an increase in the prevalence of physical activity among adolescents in some studies (*n* = 16), but an almost equal decrease in physical activity in others (*n* = 15). Research in children and adolescents also confirms the preventive role of physical activity in obesity prevention, musculoskeletal, cardiometabolic, social and emotional health, and results have also shown a significant correlation between regular physical activity and improved health-related quality of life^9–11^. Adolescents who do not engage in the amount and intensity of physical activity recommended by the WHO are more likely to experience problems with their mental health and well-being^12–14^. A literature review by Biddle et al.^15^ also showed an association between physical activity and mental health in young people, and a causal relationship was also found for cognitive functioning. In a study by Jussila et al.^16^ (*n* = 32,829) of Finnish 8th and 9th graders (M = 15.4 years), 30 min of leisure-time physical activity per week was associated with a 17% lower likelihood of chronic stress symptoms. Regular physical activity can increase self-esteem, life satisfaction and social well-being, improve sleep quality and reduce risk factors for depression^17,18^.

According to Antonovsky’s model of salutogenesis, health is much more than low levels of risk factors. Antonovsky believes that the origin of health is a stress-oriented concept that focuses on resources, leading to a greater sense of coherence that maintains and improves health and helps to choose preventive behaviours. The sense of coherence is a cognitive-level construct of comprehensibility, a behavioural-level manifestation of manageability, and a motivational-level grasp of meaningfulness. It enables individuals to process stimuli from their external environment in an organised manner, preventing them from perceiving these stimuli as inexplicable; thus, difficulties become solvable, resources can be mobilised, and addressing the problem appears worthwhile. Health depends on an individual’s ability to cope with external forces, circumstances, threatening environmental influences, stressors and to maintain a balanced sense of subjective health^19–21^. Sense of coherence (and its extent) is a key determinant and explanatory factor of health status, which is closely related to health and the positive self-perception of health.

Based on the research of Skrabski et al.^22^ the sense of coherence has shown a strong correlation with both physical and mental health, regardless of gender, age, or educational attainment. Thus, the sense of coherence has proven to be a crucial predictor of maintaining physical and mental well-being. The ability to utilise and apply available resources, reflected in the sense of coherence, can serve as a source of health, which is more significantly developable at a young age. Therefore, understanding the relationship between physical activity and the sense of coherence is of particular importance. As the sense of coherence can aid in the adoption of preventive behaviours, it may also influence the choice of physical activity. In the study by Csima et al.^23^ a higher sense of coherence was associated with a higher level of regular physical activity.

The longitudinal study by Szovák et al.^24^ examined the impact of weekly supervised sports activities, guided by a personal trainer, on changes in the sense of coherence among individuals aged 18 to 61; their findings revealed a significant increase in the sense of coherence, regardless of gender. Therefore, the aim of this study was to examine the relationship between students’ sense of coherence and physical activity. Another objective of the present study was to assess the physical activity of high school students and understand its impact on subjective general health, vitality and mental health.

To ensure methodological rigor, the present study employed standardized and psychometrically validated instruments. Physical activity-related questions were adapted from the Health Behaviour in School-aged Children (HBSC) study, based on WHO recommendations, and have been widely used in international adolescent health research^7^. Subjective health perceptions were measured using the EQ-VAS health thermometer and selected subscales (general health, vitality, and mental health) from the SF-36 Health Survey. The SF-36 has demonstrated high internal consistency (Cronbach’s α > 0.80) and well-established construct validity in both clinical and general populations, including adolescents^30,31^.

The EQ-VAS has also shown acceptable reliability and convergent validity as a sensitive indicator of self-perceived health status^43^. To assess sense of coherence, the 13-item version of Antonovsky’s SOC scale was applied, which has demonstrated strong internal reliability (α = 0.70–0.92) and factorial validity across various age groups. The Hungarian adaptation of the SOC scale has also been validated in adolescent populations^32^. The use of these validated tools enhances the reliability and interpretability of the study findings.

## Methods

The quantitative, cross-sectional research was conducted in public educational institutions in Vas and Zala Counties, located in the Western Transdanubian region of Hungary, and included students from full-time secondary schools. Data collection took place between April 24 and October 15, 2024. To be included in the sample, participants had to be enrolled in grades 9 to 12 of secondary education. In order to maintain sample homogeneity, vocational training schools were excluded, and only general secondary school classes were involved. A pilot study was carried out in one secondary school class (*n* = 28), and the questionnaire was finalized based on the feedback received. The study was implemented with the approval of the principals of the participating schools and based on passive parental consent, in accordance with recognized ethical standards in educational research^41,42^.

### Sampling

The inclusion criterion for the sample was students aged 15–19 attending a full-time four-year secondary school, and the exclusion criterion was students studying as private students.

The research was a one-time, exploratory study. Each student participated in completing the measurement tool only once, with the aim of understanding the impact of physical activity and exploring its relationship with the sense of coherence as an influencing factor. In our research instrument, we have adopted items from the questionnaires of representative national and international surveys, so our results are comparable with these data and it was not necessary to create a sub-sample or control group for the purposes of this research. To ensure efficient data collection, a convenience sampling approach was used the primary sampling units were randomly selected classes (using the systematic random method), rather than individual students.

### Measuring instrument used

The paper-based questionnaire included self-edited questions, along with socio-demographic variables and standardised scales. The physical activity-related questions in the questionnaire were taken from the instrument used in the HBSC research. The weekly occurrence of physical activity, as one of the most important health-promoting behaviours, was measured based on the WHO recommendation for children and adolescents to engage in at least 60 min of moderate-intensity physical activity per day^25^.

To measure subjective health perceptions, the EQ-VAS health thermometer and the Health Survey Short Form SF-36 Questionnaire were used in this study. On a Visual Analogue Scale (VAS), respondents were asked to mark their current health status on a line from perfect health (100 points) to worst health (0 points), a so-called health thermometer, and to indicate their relationship to the endpoints of the scale^26,27^.

The 36-Item Short Form Survey (SF-36) is widely applicable for monitoring the health status of the population aged 14 and above, evaluating the results of health care treatments, estimating the burden of disease^28,29^. The SF-36 is a multi-item scale measuring eight health concepts: (1) physical functioning (limitations in physical activities because of health problem); (2) social functioning (limitations in social activities because of physical or emotional problems); (3) physical role (limitations in usual role activities because of physical health problems); (4) bodily pain; (5) general mental health (psychological distress and well-being); (6) emotional role (limitations in usual role activities because of emotional problems); (7) vitality (energy and fatigue); (8) general health perceptions and, in addition, by summing the eight dimensions, a single health outcome. A higher score indicates better health^28,30,31^.

The target group of the research included healthy 15- to 19-year-old secondary school students; therefore, from the previously listed question groups, the measurement tool used in this research included the following items from the SF 36 questionnaire: general health perception, vitality and mental health^31^. General health perceptions were measured by five questions, vitality and mental health by transformed scale scores calculated from the combined scores of 4–4 questions, thus ranging from 0 to 100. A shortened standardised questionnaire of 13 questions was used to measure the sense of coherence which was validated in Hungarian by Balajti et al.^32^ Respondents were asked to indicate their level of agreement with the statements on a 7-point Likert scale, ranging from very rarely/never to very often/always, within a scale range of one to seven. Based on the total number of responses to the 13 items, the possible score for sense of coherence ranged from 0 to 91. The 13-item sense of coherence scale for adults is also applicable to children aged 12 years and older^33^.

### Sample

**Fig. 1:**
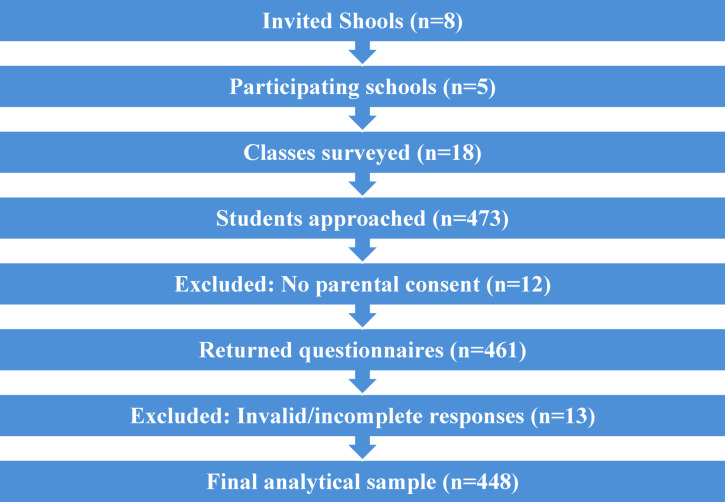
Flow diagram of school recruitment, student participation, and sample selection.

A total of 8 schools were contacted to participate in the study, of which 5 agreed to take part. In these schools, a total of 18 classes were surveyed, and the questionnaire reached 473 students. Twelve parents returned the passive consent form, resulting in the exclusion of these students from the data collection process. A total of 461 questionnaires were therefore completed and returned. During the computer-assisted data cleaning process, 13 questionnaires were excluded due to more than 50% missing answers or clearly unserious responses. As a result, the final analytical sample consisted of 448 students. (Fig. 1.)

### Applied statistical tests, software

For the analysis was used SPSS Statistics 29 software. Normality was tested using the Kolmogorov-Smirnov test and then, to determine differences between groups, a t-test and ANOVA were used for parametric variables, while the Mann-Whitney and Kruskal-Wallis tests were used for non-parametric variables (*p* < 0.05).

### Ethical compliance

The study was conducted in accordance with the Declaration of Helsinki, and was approved by the Regional Research Ethics Committee of the University of Pécs (approval code: RKEB/IKEB 24/2024, issued on 18 June 2024). Initial contact with the participating schools was established in April 2024. Recruitment of student participants began in September 2024, and data collection was completed by 15 October 2024.

Permission was obtained from the heads of the participating institutions. The research was conducted ethically; the results are reported honestly, the submitted work is original and free from (self-)plagiarism, and authorship reflects each contributor’s role.

Measurement instruments were completed anonymously, and no personal identifiers were collected. After data processing, results were reported in aggregate form, with no information disclosed about individual students, classes, or schools. Informed consent was obtained from the parents or legal guardians via a passive consent procedure. Accordingly, students whose parents did not approve participation did not return the questionnaire.

## Results

### Description of the socio-demographic/biographical characteristics of the sample

In the survey of 10th, 11th and 12th grade secondary school students in Western Transdanubia, the mean age of the respondents was 16.87 years (SD: 0.82), two-thirds were female, more than half lived in a town and two-fifths lived in the same municipality as the school. The majority of the sample (69.87%) lived in a full family. 28.79% (129) of the families had one child and 22.1% (99) had three or more children.

### Examining the relationship between physical activity and subjective perceptions of health

The HBSC (Health Behaviour of School-Aged Children) survey focuses on understanding the health status of adolescents, examining the factors that individually and collectively influence the health of children and teenagers, providing a comprehensive overview of boys’ and girls’ health, well-being, and health-related behaviours. A question assessing the frequency of physical activity was adapted from the measurement tool of this survey. Students were asked to reflect on the past seven days and count how many days they engaged in at least 60 min of vigorous physical activity. 60 min of exercise per day was considered to be an adequate amount of physical activity. Categorisation was based on the Health Behaviour in School-aged Children (HBSC) study classification (‘adequate’ if 60 min of physical activity were done every day of the week, ‘nearly adequate’ if at least 5–6 days per week, ‘little’ if 3–4 days per week, and ‘very little’ if 2 days or less per week). 22.2% of the young people who responded were classified as doing an adequate amount of physical activity, 29.02%−29.02% as doing nearly adequate or little physical activity, and 19.76% as doing very little physical activity.

The qualitative assessment of physical activity was measured using two questions, determining how many times per week and for how long an individual engaged in exercise that involved sweating.

Based on this, with regard to the quality of physical activity (its intensity and duration), engaging in sweat-inducing physical activity for a total of at least 3 h on at least three days per week is considered adequate, a criterion met by 64.1% of the sample but 9.6% of the sample did not engage in any vigorous physical activity at all.

The health status of the respondents was measured using the EQ-VAS health thermometer. The mean value of the actual health perception was 79.16 on a scale of 100 (SD: 16.5, max: 100, min: 10). The students’ health status was also assessed using the standardised health assessment instrument SF-36. The reliability values of the scales were adequate (general health Cronbach’s alpha = 0.77, mental health Cronbach’s alpha = 0.83, vitality Cronbach’s alpha = 0.81).

In this sample, the mean for general health perception was M: 74.94 (SD: 17.31), for mental health M: 68.33 (SD: 19.06) and for vitality 50.47 (SD: 20.59).

**Fig. 2:**
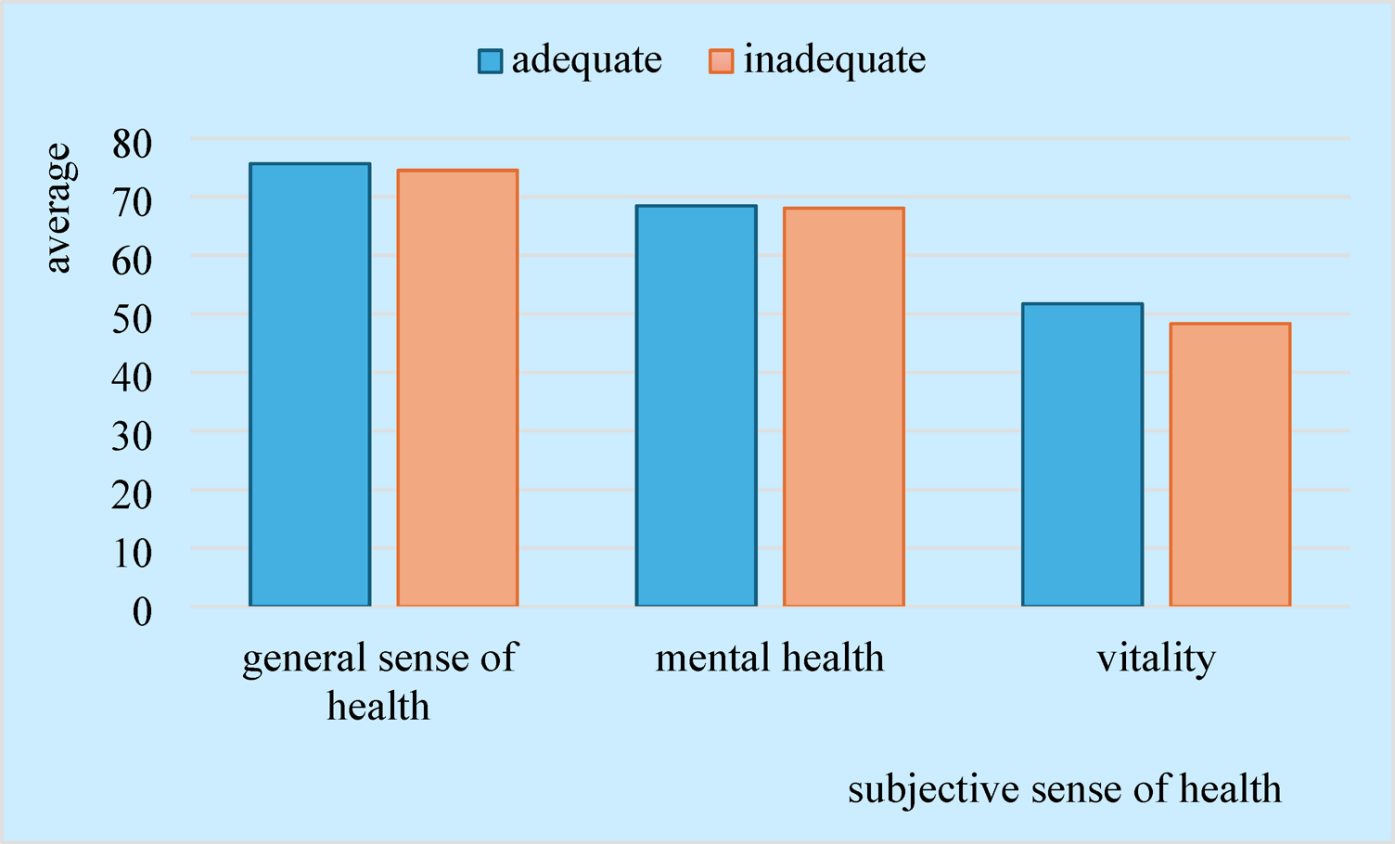
Mean self-rated health scores based on qualitative indicator of physical activity, *n* = 445.

Based on the quality of physical activity, the study sample was divided into two groups (adequate, inadequate) and the relationship between physical activity and subjective health perception was measured. The difference in the mean self-rated health scores of the groups was tested using a t-test. The mean scores for general health perception were not significantly different (t(446) −0.676 *p* = 0.52) for qualitatively adequate (M = 75.68) and inadequate physical activity (M = 74.52). Mean values for the other subjective health indicators examined in the groups were also nearly identical for mental health (t(445) 0.149 *p* = 0.882), vitality (t(446) 1.677 *p* = 0.094) (Fig. 2).

### Examining the relationship between quantitative indicators of physical activity and health perceptions

**Fig. 3:**
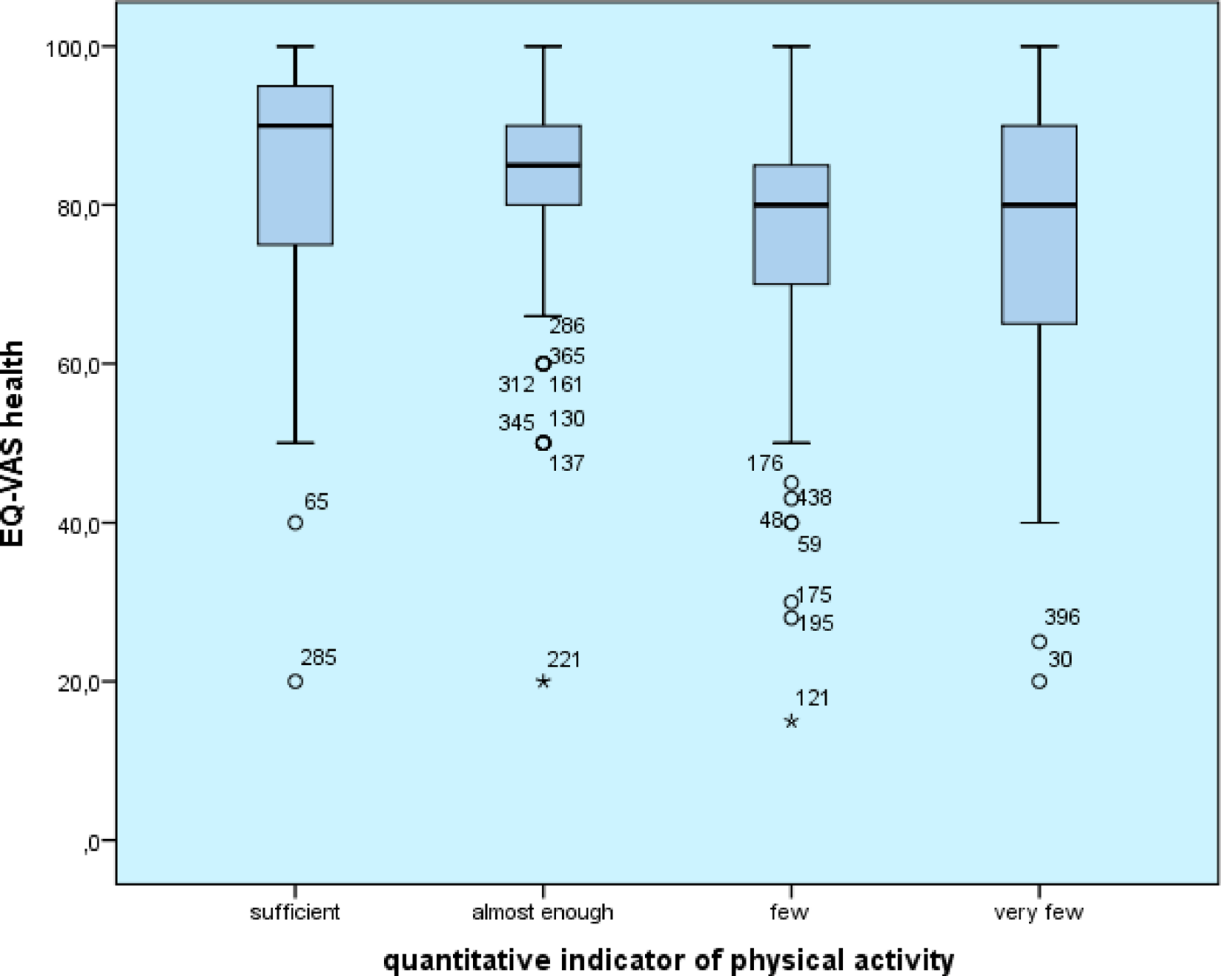
Mean self-rated health scores based on quantitative indicator of physical activity, *n* = 416.

In this study, changes in physical activity were also examined in relation to students’ current health perceptions as measured by the Visual Analogue Scale. A Kruskal-Wallis test was used to test the effect of physical activity on self-rated health, based on the Kolmogorov-Smirnov test of normality of the data obtained from the EQ visual analogue scale. Based on the analysis, a significant difference in the variation of physical activity quantity was demonstrated for self-reported health perception (H(3,416)24.34 *p* < 0.001) (adequate physical activity: M = 84.45; nearly adequate: M = 82.4; low: M = 76.58; very low: M = 76.36), which was not significant for the qualitative indicator of physical activity (U = 22936.0, z = −0.067, *p* = 0) (qualitatively adequate M = 74.52; inadequate physical activity M = 75.68). As the amount of physical activity increased, the mean scores for the other domains of subjective health perception also increased, most significantly for vitality (adequate physical activity M = 55.46; nearly adequate M = 54.62; little M = 46.11; very little M = 45.84). When examining vitality, a significant difference (M = 9.62 points) was found between the two extreme groups in terms of frequency of physical activity for the ‘adequate’ and ‘very little’ categories (F(3,417)7.371 *p* < 0.001). Based on the results, the young people who reported the most positive general health (M = 79) and mental health (M = 73.93) were those who engaged in adequate physical activity (at least 60 min of physical activity per day, every week). Results show that with increasing frequency of weekly physical activity, mean scores for general health (F(3,417)3,146 *p* = 0.025) and mental health (F(3,417)5,88 *p* = 0.001) also increased significantly (Fig. 3).

### Examining the relationship between physical activity and a sense of coherence

The analysis also examined the relationship between physical activity as a preventive behaviour and the sense of coherence. It was hypothesised that the frequency and intensity of leisure-time physical activity would be influenced by the individual’s sense of coherence. The exact value of the sense of coherence —the point at which the measured data can be considered a risk factor—cannot be determined. At an individual level, the sense of coherence can only be determined at a particular point in time.

**Fig. 4:**
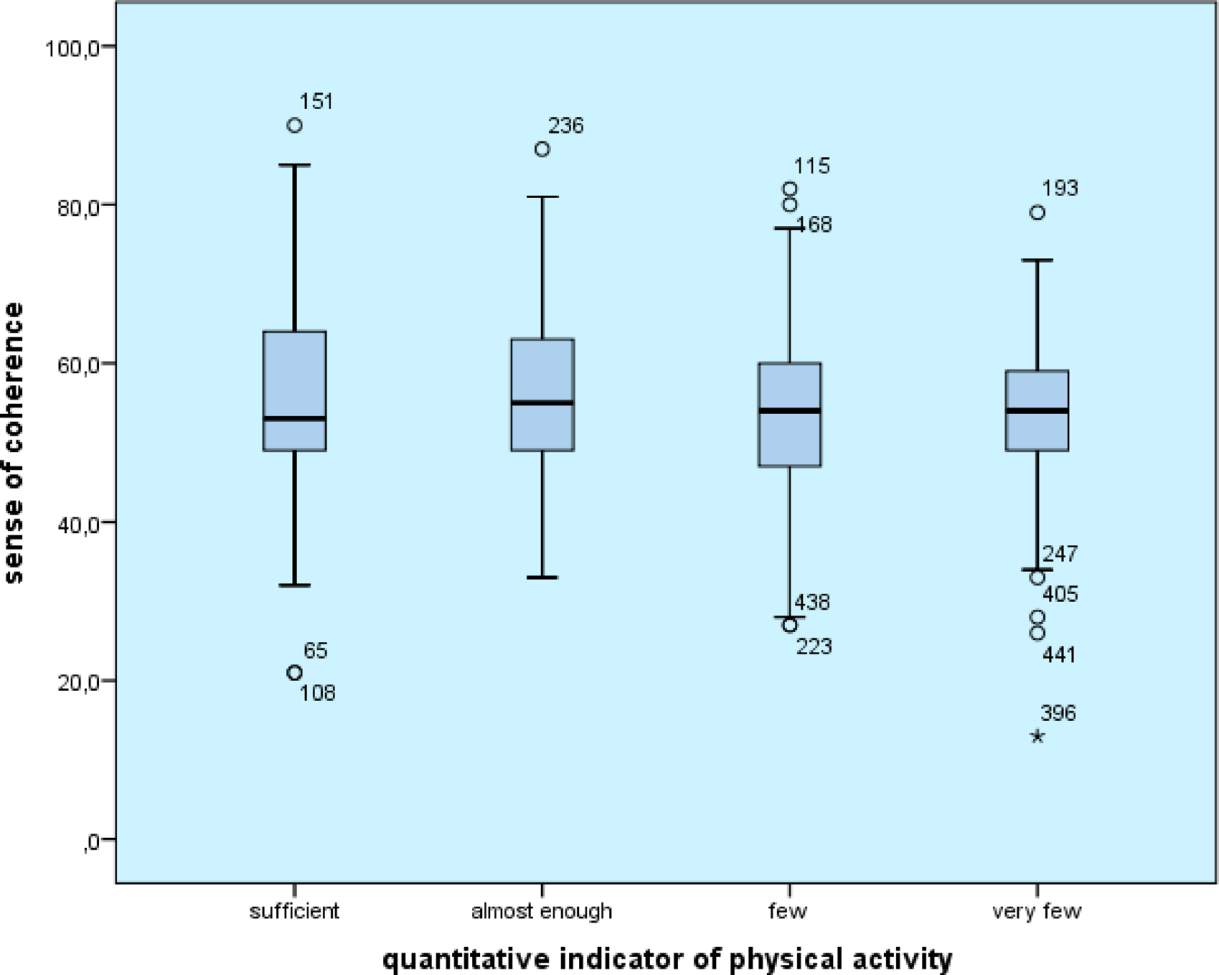
Relationship between sense of coherence and frequency of physical activity (*n* = 416).

In this survey, the mean score for sense of coherence was 54.38 (SD: 11.35, max: 90, min: 13) based on the responses of secondary school students aged 15–19. The reliability value of the 13-item scale was adequate (Cronbach’s alpha = 0.808). To determine the correlation between the sense of coherence and the frequency of physical activity, the Kruskal-Wallis test was used after the normality test, and the results showed that the value of the sense of coherence was significantly different according to the amount of physical activity of the students (H(3,416)9,821 *p* = 0.02) (adequate physical activity: M = 55.96; nearly adequate: M = 56.48; low: M = 52.82; very low: M = 53.09). However, after calculations with the Mann-Whitney test, the sense of coherence did not significantly differentiate the quality indicator of students’ physical activity (U = 22819.5 z=−0.107 *p* = 0.195) qualitatively adequate: M = 54.43; inadequate physical activity: M = 54.26) (Fig. 4).

When interpreting the results, the limitations of the research must also be taken into account. Although the schools included in the study are homogeneous in terms of school type, they cannot be considered representative, and further studies in other types of schools and on a representative sample are needed to make the results generalisable. In addition, the respondents are not completely unrelated to each other, as school classes participated in the survey. It should also be noted that the quantity and quality of physical activity and the perception of health status were based on self-reports by the students.

## Discussion

Physical activity remains one of the most crucial factors influencing individual and population-wide well-being, health, and quality of life. Considering the recommendations of the World Health Organization^25^ the level of physical activity is still inadequate in many social groups, as demonstrated in the case group of this study. Looking at the amount of physical activity in the current study, one in two students engaged in an adequate or nearly adequate amount of physical activity, 5–7 times per week, but two in ten students had very little weekly physical activity. The quality of physical activity was only adequate for two out of three students. Overall, the presented quantitative and qualitative indicators of leisure-time physical activity are suboptimal, which is consistent with international and national research findings among adolescents^7,8^. Sedentary lifestyle is a global problem in developed countries, posing a challenge in terms of health promotion, health preservation and economic aspects. In terms of the health status of the responding students, the overall health perception is considered adequate compared to previous research. In the sample, the mean scores were as follows: general health perception M = 74.94 (SD = 17.31), mental health M = 68.33 (SD = 19.06), and vitality M = 50.47 (SD = 20.59). The general health perception of the respondents was nearly identical to the average normative value for the healthy population under 18 years of age in Hungary (M = 74)^34^.

The mean normal values for vitality and mental health of the healthy Hungarian population under 18 years of age in 1999 were 79 and 79, which is much lower for the current study population^34^. However, evidence from the 2018 international HBSC survey shows a significant downward trend in mental health scores for school-aged youth compared to data from 2002, 2006, 2010 and 2014^7^.

The results indicate that the subjective indicators of general health status did not significantly deviate from the averages reported in previous research. However, the decline in mental health levels further justifies the necessity of this survey and the efforts aimed at its improvement.

The amount of physical activity was positively correlated with measured self-rated health indicators, which has been confirmed by previous research as well^12–15^. Engaging in regular physical activity can be a protective factor. The results confirm the importance of physical activity as a health-promoting health behaviour for self-reported health perceptions. In order to maintain and improve somatic-mental health and well-being, strategies to increase the frequency and intensity of regular physical activity among adolescents and young people should be implemented to promote exercise, which would also lead to a reduction in healthcare-related expenditures^35,36^. Regular physical activity is associated with better subjective health, which can also prevent the development of certain diseases. The research findings confirm that regular physical activity helps prevent diabetes, metabolic syndrome, cardiovascular diseases, high blood pressure, obesity, osteoporosis, cerebrovascular diseases, ischaemic heart disease, and certain types of cancer^37,38^. Regular physical activity, performed correctly, becomes part of everyday life from childhood, with positive effects on health in adulthood.

In the meta-analysis-based research conducted by Eriksson and Lindstrom^19^ the mean values of the sense of coherence ranged from 35.39 (SD = 0.1) to 77.6 (SD = 13.8).

A study conducted on a Hungarian sample of 16- to 17-year-olds (*n* = 1127) found an average sense of coherence score of 53.4^39^.

Based on these data, the measured mean value of the sense of coherence (*M* = 54.8) in this survey fell within a similar range to the previously reported values.According to the results of this research, the sense of coherence plays a role in the changes in the regularity of physical activity, and a linear stochastic relationship between the variables was demonstrated, which was also proved by a study among adolescents in Kaposvár, Hungary, where a greater sense of coherence was measured among youths who played sports every day of the week outside of physical education classes^23^. The research conducted by Jeges and Varga^40^ also confirmed the indirect effect of physical activity on strengthening the sense of coherence The foundations for long-term health can be established in childhood and it is therefore very important to reduce and stop negative health behaviours and to strengthen and increase positive ones. According to the salutogenetic model, developing a sense of coherence through general resources is of utmost importance. The regularity of leisure-time physical activity can also be enhanced through general resources. In the teenage years, the role of peer relationships is particularly important in developing a healthy lifestyle.

## Conclusion

This study confirmed that regular physical activity, especially when performed daily and with sufficient intensity, is associated with better self-perceived general health, vitality, and mental health among adolescents. A higher sense of coherence was also linked to greater physical activity engagement, reinforcing the relevance of Antonovsky’s salutogenic model. These findings support the promotion of physical activity as a key preventive behavior and underline the importance of strengthening internal and environmental resources to support adolescent health.

## Data Availability

The datasets used and/or analysed during the current study available from the corresponding author on reasonable request.
